# A Risk Prediction Flowchart of Vancomycin-Induced Acute Kidney Injury to Use When Starting Vancomycin Administration: A Multicenter Retrospective Study

**DOI:** 10.3390/antibiotics9120920

**Published:** 2020-12-18

**Authors:** Takayuki Miyai, Shungo Imai, Hitoshi Kashiwagi, Yuki Sato, Shota Kadomura, Kenji Yoshida, Eri Yoshimura, Toshiaki Teraya, Takashi Tsujimoto, Yukari Kawamoto, Tatsuya Itoh, Hidefumi Ueno, Yoshikazu Goto, Yoh Takekuma, Mitsuru Sugawara

**Affiliations:** 1 Graduate School of Life Science, Hokkaido University, Sapporo 060-0810, Japan; t-miyai@eis.hokudai.ac.jp; 2 Faculty of Pharmaceutical Sciences, Hokkaido University, Sapporo 060-0812, Japan; h-kashi@pharm.hokudai.ac.jp (H.K.); yukis@pharm.hokudai.ac.jp (Y.S.); msuga@pharm.hokudai.ac.jp (M.S.); 3 Department of Pharmacy, Japan Community Healthcare Organization Sapporo Hokushin Hospital, Sapporo 004-8618, Japan; kadomura-shota@hokushin.jcho.go.jp (S.K.); itoh-tatsuya@hokushin.jcho.go.jp (T.I.); 4 Department of Pharmacy, Sunagawa City Medical Center, Sunagawa 073-0196, Japan; kenji.yoshida.8314@gmail.com (K.Y.); yoshimukedama@gmail.com (E.Y.); hueno@med.sunagawa.hokkaido.jp (H.U.); 5 Department of Pharmacy, Sapporo City General Hospital, Sapporo 060-8604, Japan; toshiaki.teraya@city.sapporo.jp (T.T.); takashi.tsujimoto@city.sapporo.jp (T.T.); yukari.kawamoto@city.sapporo.jp (Y.K.); yoshikazu.goto@city.sapporo.jp (Y.G.); 6 Department of Pharmacy, Hokkaido University Hospital, Sapporo 060-8648, Japan; y-kuma@pharm.hokudai.ac.jp; 7 Global Station for Biosurfaces and Drug Discovery, Hokkaido University, Sapporo 060-0808, Japan

**Keywords:** vancomycin, therapeutic drug monitoring, decision tree analysis, acute kidney injury

## Abstract

We previously constructed a risk prediction model of vancomycin (VCM)-associated nephrotoxicity for use when performing initial therapeutic drug monitoring (TDM), using decision tree analysis. However, we could not build a model to be used at the time of initial administration due to insufficient sample size. Therefore, we performed a multicenter study at four hospitals in Japan. We investigated patients who received VCM intravenously at a standard dose from the first day until the initial TDM from November 2011 to March 2019. Acute kidney injury (AKI) was defined according to the criteria established by the “Kidney disease: Improving global outcomes” group. We extracted potential risk factors that could be evaluated on the day of initial administration and constructed a flowchart using a chi-squared automatic interaction detection algorithm. Among 843 patients, 115 (13.6%) developed AKI. The flowchart comprised three splitting variables (concomitant drugs (vasopressor drugs and tazobactam/piperacillin) and body mass index ≥ 30) and four subgroups. The incidence rates of AKI ranged from 9.34 to 36.8%, and they were classified as low-, intermediate-, and high-risk groups. The accuracy of flowchart was judged appropriate (86.4%). We successfully constructed a simple flowchart predicting VCM-induced AKI to be used when starting VCM administration.

## 1. Introduction

Acute kidney injury (AKI) is a clinical syndrome characterized by rapidly declining renal function and renal tissue damage [1]. AKI is confirmed when any of the following criteria are met: (1) increase in serum creatinine (Scr) concentration ≥ 50% within 7 days (2) increase in Scr concentration ≥ 0.3 mg/dL within 2 days, and (3) urine output less than 0.5 mL/kg/h for 6–12 h. These criteria were established by the Kidney disease: Improving global outcomes (KDIGO) group [2]. However, even mild AKI can result in increased mortality and prolonged hospitalization [3,4]. Thus, early diagnosis of AKI is important for improving patient prognosis.

Vancomycin (VCM) is a glycopeptide antibiotic used to treat methicillin-resistant *Staphylococcus aureus* infections [5]. However, nephrotoxicity is a major side effect of VCM, as evidenced by the 5–43% incidence of VCM-induced nephrotoxicity. Most episodes occurred during 4–17 d of VCM therapy [6]. In preclinical studies, VCM-induced AKI is presumed to be a result of the accumulation of the drug in proximal tubule cells, which triggers cellular oxidative stress and apoptosis. However, the underlying mechanism of VCM-induced AKI remains poorly understood [7]. To prevent a deteriorated prognosis, early detection of nephrotoxicity is crucial for patients treated with VCM [6]. Risk factors associated with VCM-induced nephrotoxicity have been reported in several studies, and clinicians and pharmacists should pay attention to patients with these risk factors [8,9,10,11,12,13,14,15,16].

Previously, we constructed a risk prediction model of VCM-induced nephrotoxicity [17]. The model was based on data from 592 patients who underwent therapeutic drug monitoring (TDM) and was constructed using decision tree (DT) analysis. DT analysis is commonly used to predict dependent variables from independent variables and to explain the relationship between independent variables [18]. It involves composing a flowchart-like tree framework by repeating the process that classifies data according to specific conditions. Therefore, by using our model, users can easily estimate combinations of factors that may increase the risk of VCM-induced nephrotoxicity [17]. Additionally, the accuracy of this DT-based model was equivalent to a conventional logistic regression model. However, our model included factors that could not be obtained at the time of use (i.e., when initial TDM).

Next, we constructed a risk prediction model for use at the time of the initial TDM [19]. The model was based on data from 402 patients who were administered VCM for 7–14 d and underwent TDM. The possibility of clinical use has been shown by our model using only variables that could be obtained at the time of initial TDM [19]. However, the risk prediction model for use when initiating VCM administration could not be constructed. Constructing a risk prediction model for when the VCM therapy is initiated requires patients administered standard doses; however, pharmacists usually determine initial doses based on guidelines or several studies [20,21]. Moreover, our previous study could not include enough patients who were administered standard doses. In addition, although prolonged VCM therapy is a risk factor for nephrotoxicity [13,22], the administration period is usually undecided at the time of initial VCM administration. Recognizing AKI as early nephrotoxicity after administration is important for setting the appropriate initial dose [2,5,20].

Therefore, we performed a multicenter study to construct a simple flowchart that can be used by medical staff to predict VCM-induced AKI before VCM therapy.

## 2. Results

### 2.1. Characteristics of Patients 

**Figure 1:**
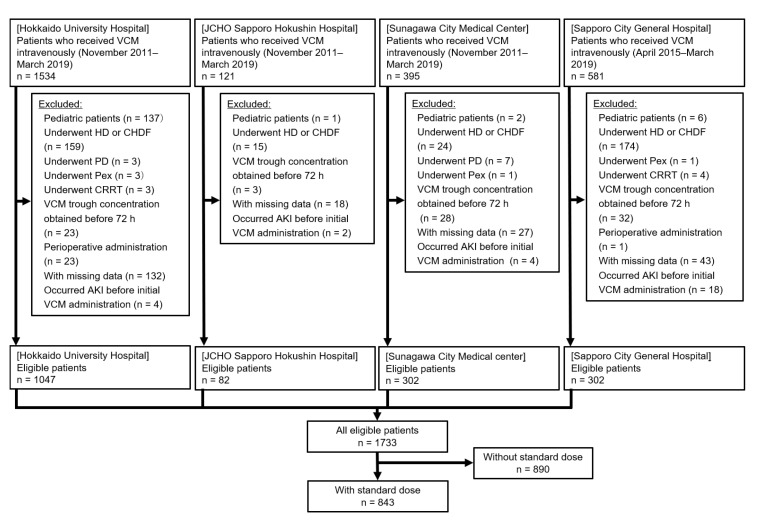
Flowchart of patients included in this study. The standard dose was defined within the recommended dose ± 250 mg in reference to Thomson et al.’s study [21] (Table 3) (for example, in patients with creatinine clearance > 110, Thomson et al. recommended 1500 mg every 12 h, whereas we defined the standard dose as 2500–3500 mg per day). Abbreviations: JCHO, Japan Community Healthcare Organization; VCM, vancomycin; HD, hemodialysis; CHDF, continuous hemodiafiltration; PD, peritoneal dialysis; Pex, plasma exchange; CRRT, continuous renal replacement therapy; AKI, acute kidney injury.

Out of 2631 patients, 843 were included in this study. Of the included patients, 115 (13.6%) developed VCM-induced AKI (Figure 1).

A comparison of patient characteristics revealed significant differences in the following 17 characteristics: body weight, body mass index (BMI), blood urea nitrogen (BUN); initial administration, initial TDM, maximum), Scr (initial administration, initial TDM, maximum), VCM daily dosage after initial TDM, duration of therapy, initial VCM trough concentration, residence in the intensive care unit (ICU), underlying heart disease, and several concomitant medications (furosemide, amphotericin B (AMPH-B), vasopressor drugs, and tazobactam/piperacillin (TAZ/PIPC)) (Table 1).

### 2.2. Logistic Regression Analysis

In univariate logistic regression analysis, the following parameters were *p* < 0.2: underlying heart disease, several concomitant medications (furosemide, AMPH-B, vasopressor drugs, and TAZ/PIPC), and BMI ≥ 30. In multiple logistic regression analysis, *p* < 0.05 was shown in several concomitant medications (furosemide, AMPH-B, vasopressor drugs, and TAZ/PIPC) (Table 2).

### 2.3. DT Analysis

**Figure 2:**
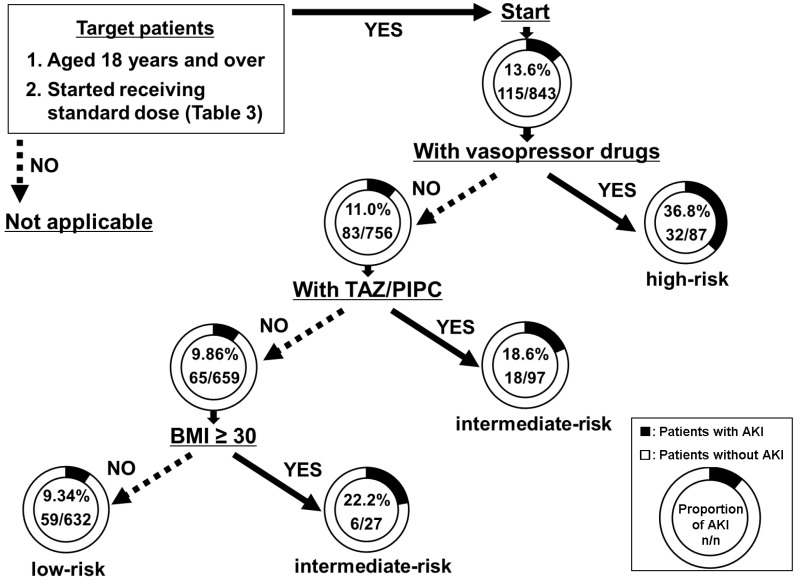
Flowchart for the risk prediction of VCM-induced AKI. A flowchart was proposed for predicting the risk of VCM-induced AKI at the time of administering the standard dose when initiating VCM therapy. Target patients are “aged 18 years and over” and “started standard dose of VCM”. Subgroups of the flowchart were categorized by the proportion of VCM-induced AKI as follows: (**1**) low-risk group (<10%), (**2**) intermediate-risk group (10–25%), and (**3**) high-risk group (>25%). Abbreviations: AKI, acute kidney injury; VCM, vancomycin; TAZ/PIPC, tazobactam/piperacillin, BMI: body mass index.

A flowchart predicting the risk of VCM-induced AKI was constructed using DT analysis. Patients aged 18 years and over, who started receiving standard dose, were included for the construction of the flowchart. This flowchart included three splitting variables and four subgroups. Splitting variables were chosen from risk factors strongly related to VCM-induced AKI (Figure 2). Vasopressor drugs were chosen as the first splitting variable. With vasopressor drugs, the proportion of AKI was 36.8%, and patients receiving them were classified into the high-risk group. Without vasopressor drugs, the proportion of AKI was 11.0% and TAZ/PIPC was chosen as the second splitting variable. With TAZ/PIPC, the proportion of AKI was 18.6% and patients receiving TAZ/PIPC were classified as being at intermediate risk. Without TAZ/PIPC, the proportion of AKI was 9.86% and BMI ≥ 30 was chosen as the third splitting variable. With BMI ≥ 30, the proportion of AKI was 22.2% and these patients were also classified as being at intermediate risk. Without BMI ≥ 30, the proportion of AKI was 9.34% and patients were classified as being at low risk. 

### 2.4. Validation of the Model

By using the Hosmer–Lemeshow test, we determined *p* = 0.454. The misclassification risk of the flowchart was 13.9 ± 1.2% in 10-fold cross validation. The accuracies of the DT analysis and multiple logistic regression analysis were 86.4% and 86.5%, respectively.

## 3. Discussion

By using DT analysis, we successfully constructed a flowchart for predicting the risk of VCM-induced AKI (i.e., nephrotoxicity occurring within 7 days after administration) at the time of initial VCM administration. When constructing the flowchart, we excluded “VCM dose” as a risk factor. In general, overdose of VCM is a major risk factor of AKI and patients are administered “VCM dose” designed to increase clinical efficacy and decrease toxicity according to clinical guidelines or previous studies. Initial VCM doses are decided by the clinicians and pharmacists to avoid excessive response to VCM in terms of the individual patient’s characteristics such as creatinine clearance (CCr). Therefore, it is necessary to exclude the factor of “VCM dose” when constructing the risk prediction flowchart of AKI for use when deciding the initial dose. Thus, we targeted patients of “18 years and over” and those who had “started a standard dose of VCM”. We referred to a study by Thomson et al. to define the standard dose, and the margin of VCM dose was set to ± 250 mg at a time [21].

By using our flowchart at the start of VCM administration, clinicians and pharmacists can evaluate the risk of AKI in patients who are administered a standard dose of VCM. For example, when a clinician decides to administer VCM to an obese patient (for example, with BMI = 32 kg/m^2^) who does not concomitantly use vasopressor drugs and TAZ/PIPC, the patient would be predicted to be in the intermediate-risk group (22.2%) for the VCM-induced AKI, according to our flowchart. Moreover, because patient data were extracted from four Japanese hospitals, selection bias is expected to be smaller than that observed when extracting data from a single hospital.

Out of 843 patients, 115 (13.6%) developed VCM-induced AKI. The proportions of VCM-induced AKI were 10–25% in 11 of the 15 studies included in a previous systematic review [6]. Fewer patients developed AKI in our study compared to those in previous studies. This is because we targeted AKI observed within seven days after the initial VCM administration and not throughout the treatment regimen.

In the DT analysis, three splitting variables were selected from six variables that exhibited a statistical significance of *p* < 0.2 in univariate analysis. First, “concomitant with vasopressor drugs” was classified as the high-risk group (the proportion of VCM-induced AKI was 36.8%). The risk of AKI is increased by hemodynamic instability (e.g., persistent hypotension) [2]. Vasopressor drugs are recommended for maintaining renal blood flow in patients with unstable hemodynamic status. Thus, patients administered VCM concomitantly with vasopressor drugs are assumed to be at increased risk of developing VCM-induced AKI. Second, “concomitant with TAZ/PIPC” was selected as a splitting variable in the group without concomitantly administered vasopressor drugs, and the proportion of VCM-induced AKI was 18.6% (intermediate-risk group). Several studies have reported that the risk of VCM-induced AKI is increased by concomitant use of TAZ/PIPC [12,23]. Although the mechanism of this interaction has not been established, some hypotheses have been reported as follows [23,24,25]: (i) interstitial nephritis of TAZ/PIPC and acute tubular necrosis due to accumulated VCM influence each other and (ii) TAZ/PIPC decreases clearance of VCM and increases accumulation in the body. Nevertheless, it is necessary to watch out for AKI occurrences in the group administered TAZ/PIPC. Third, “BMI ≥ 30” was selected as the splitting variable in the group that was not administered concomitant vasopressor drugs and TAZ/PIPC, and the proportion of VCM-induced AKI was 22.2% (intermediate-risk group). In this study, we used “BMI ≥ 30” as an alternative indicator of “body weight > 100 kg” [5,26]. Considering the calculated dose per body weight, obese patients received an increased dose of VCM that may increase the risk of AKI. However, we targeted patients who received the standard dose according to their CCr. Patients who were administered a daily dose of ≥ 4000 mg were not included. As obesity affects the pharmacokinetic parameters of VCM [21], it may be associated with our results. Thus, frequent monitoring of VCM trough concentrations is required in patients with obesity. In future research, it is necessary to elucidate the mechanism by which obesity increases the risk of VCM-induced AKI. 

To validate the results of the DT analysis, multiple logistic regression analysis was performed. Four variables (concomitant with vasopressor drugs, AMPH-B, TAZ/PIPC, and furosemide) showed significant differences. AMPH-B and furosemide were not selected as splitting variables in the flowchart. The odds ratio of AMPH-B was 9.32 (95% confidence interval (95% CI), 2.07–41.71), which was the highest of the four variables; therefore, it strongly affects AKI. However, this high odds ratio needs to be re-validated because only eight patients were administered VCM concomitantly with AMPH-B. In fact, a previous study showed that the odds ratio of AMPH-B was 2.25 (95% CI, 1.14–4.41) [12]. The odds ratio of concomitantly administered furosemide was 1.74 (95% CI, 1.09–2.76), which was lower than that of AMPH-B and vasopressor drugs.

The accuracy and misclassification of the constructed flowchart were validated by several statistical methods. The results indicated that the obtained values were equivalent to those in our previous study. Thus, our flowchart indicated good fit [17,19]. 

Striving to reduce patient’s risk for VCM-induced AKI (e.g., clinicians and pharmacists estimating a patient’s risk) is important for preventing deterioration of patient’s prognosis (such as increases in mortality and length of stay). Our flowchart is expected to help with this. For example, for patients who are predicted as high-risk according to the flowchart, clinicians and pharmacists can respond by (i) implementing TDM frequently, (ii) monitoring the parameters of renal function, such as Scr, urine volume, and cystatin C frequently, and (iii) considering alternative drugs such as linezolid, which has a relatively small effect on renal function. 

Our study has several limitations. First, we used Thomson et al.’s study to define the standard dose, and the VCM dose was set to ± 250 mg [21]. However, it is unknown whether this criterion is reasonable. Thus, as a preliminary study, we confirmed the difference in VCM trough concentration between the following two groups for validating the standard dose: (1) one group started the VCM dose recommended by Thomson et al., and (2) another group started the VCM dose recommended by Thomson et al. ± 250 mg. Our results indicated that no significant difference (*p* = 0.137, calculated by Mann–Whitney *U* test) was observed between their VCM trough concentrations (data not shown). Thomson et al. showed that 87% of the patients achieved a ratio of area under the curve to minimum inhibitory concentrations (AUC/MIC) >400 [21]. Moreover, the new guidelines provided by the Infectious Diseases Society of America recommend AUC/MIC of 400–600 as a target that is compatible with both effectiveness and safety [5]. Therefore, the standard dose defined in this study is considered reasonable. Second, the patients’ clinical background was diverse in terms of underlying disease or concomitant drugs. However, limited variables were used to construct a flowchart in this study. Thus, constructing a flowchart that targets diverse patients requires more variables for analysis. Although our flowchart is proposed for use at the time of initial VCM administration, it does not correspond to the changing background of a patient over time. Thus, to provide clinicians with a risk prediction flowchart that is more effective, demographic and background data of more patients need to be incorporated in the construction of the flowchart. Third, almost all the eligible patients were Japanese and we could not evaluate the data for patients of different ethnicities. As ethnicity affects the proportion of AKI, the data for patients of different ethnicities need to be evaluated [1].

## 4. Materials and Methods 

### 4.1. Study Design

This multicenter retrospective observational study was conducted at Hokkaido University Hospital, Japan Community Healthcare Organization (JCHO) Sapporo Hokushin Hospital, Sunagawa City Medical Center, and Sapporo City General Hospital. Some cases of Hokkaido University Hospital from our previous study were also included [17,19].

### 4.2. Subjects

All the included patients who were administered VCM intravenously between November 2011 and March 2019 at three hospitals and from April 2015 to March 2019 at the Sapporo City General Hospital, were investigated. The target period was set considering the introduction of the electronic TDM system for VCM in the Hokkaido University Hospital (the main hospital in this study) from November 2011. All data were collected from the patients’ medical records. If a patient had been administered VCM during some other period, we collected the data for the first period only. Inclusion criteria were (i) ages ≥ 18 years, (ii) trough concentration of VCM was measured after 72 h of administration, and (iii) patients had been administered a standard dose from the first day to the initial TDM. Exclusion criteria were (i) patients who underwent hemodialysis, continuous hemodiafiltration, peritoneal dialysis, plasma exchange, and renal replacement therapy; (ii) AKI occurrence before VCM administration; (iii) perioperative period; and (iv) patients with missing data. In accordance with Thomson et al. [21], the standard dose was defined within the recommended dose ± 250 mg (Table 3).

### 4.3. Data Collection

Potential risk factors for VCM-induced AKI were extracted based on the following parameters that could be evaluated on the day of initial VCM administration [8,9,10,11,12,13,14,15,16]: BMI, CCr, underlying heart disease, type of infection of infective endocarditis, and concomitant medications (non-steroidal anti-inflammatory drugs (NSAIDs), furosemide, aminoglycosides, AMPH-B, TAZ/PIPC, vasopressor drugs, nitric acid-based medicines, and tacrolimus). Vasopressor drugs included etilefrine, noradrenaline, olprinone, milrinone, dopamine, and dobutamine. Age ≥ 52 years has been reported as a risk factor for VCM-induced AKI [13]. However, because the patient’s age is correlated with CCr, we did not include it as a potential risk factor. 

Although residence in ICU has been reported as a risk factor [15,16], patients in the ICU often have other risk factors such as concomitant use of furosemide and vasopressor drugs. Therefore, we excluded ICU stay from the potential risk factors for VCM-induced AKI. Moreover, a body weight of 100 kg and over was also reported as a risk factor. As this study included a few patients weighing over 100 kg [16], BMI ≥ 30 kg/m^2^ was used as an alternative indicator. A BMI of 30 kg/m^2^ indicates obesity as defined by the World Health Organization [26]. Moreover, for evaluating patient characteristics, data regarding the patient’s age, sex, height, body weight, initial VCM trough concentration, Scr (at the time of initial VCM administration, initial TDM, and maximum), BUN at the same time as Scr, VCM daily dose (at the time of initial administration and after TDM) with loading dose, residence in ICU, duration of VCM therapy, and the day of initial TDM and underlying diseases (diabetes, hypertension, and cancer), were extracted. The loading dose was defined as the initial dose, which was 1.25 times the maintenance dose [19].

To calculate CCr at the time of the initial VCM administration, we used the Cockcroft-Gault equation [27]. If a patient’s BMI was greater than 25, adjusted weight (ABW) was used. ABW was calculated using the following formula [19]: ABW = ideal body weight (IBW) + 0.4 (actual body weight − IBW), where IBW (men) = 50 + 2.3 (inches above 60) and IBW (women) = 45 + 2.3 (inches above 60).

### 4.4. Definition of VCM-Induced AKI

Based on KDIGO classifications, VCM-induced AKI was defined as “an increase in Scr of 50% within 7 d” or “0.3 mg/dL within 48 h” from the time of initial VCM administration [2]. As we could not collect the data, we did not define AKI based on the urine output.

### 4.5. Statistical Analysis and Construction of a Flowchart

Patient characteristics were compared between two unpaired groups, and all tests of significance were two-tailed. Continuous variables were compared using the Mann–Whitney *U* test. Categorical variables were compared using Pearson’s chi-squared test or Fisher’s exact test. Fisher’s exact test was used to include one or more cells with an expected value of <5 on a 2 × 2 contingency table.

In univariate logistic regression analysis, the presence of AKI was used as an independent variable and potential risk factors were used as dependent variables. BMI and CCr that were extracted as continuous variables were converted to categorical variables based on previous reports [13,16]. Independent variables showing *p* < 0.2 in univariate analysis were used as independent variables in the multiple logistic regression analysis and DT analysis.

By using DT analysis, we constructed a flowchart predicting the risk of VCM-induced AKI. DT analysis was performed in the same way as in our previous studies that used the chi-squared automatic interaction detection (CHAID) algorithm [17,19]. The CHAID algorithm helps in constructing a flowchart using the selected significant independent variables in a chi-squared test. Constructing the flowchart was stopped when blanching three times or when parent nodes included under 20 subjects or child nodes included under 10 subjects [28].

As in our previous studies, subgroups of the flowchart were categorized by the proportion of VCM-induced AKI as follows: (i) low-risk group (<10%), (ii) intermediate-risk group (10–25%), and (iii) high-risk group (>25%) [17,19].

All statistical analyses and construction of the flowchart were performed using SPSS Statistics Version 25 (IBM, Tokyo, Japan).

### 4.6. Validation of the Model

In this study, the model was validated in the same way as in our previous studies [17,19]. The Hosmer–Lemeshow test was used to evaluate the fitness of the logistic regression model. When *p* > 0.05 was obtained in the Hosmer-Lemeshow test, we adjudged the logistic regression model to be a good-fitting one [29]. To evaluate the misclassification risk of the flowchart constructed by DT analysis, 10-fold cross validation was performed as follows. The original sample was first partitioned into 10 equal-sized subsamples. One sample was used to evaluate the training data constructed using the other samples. This process was then repeated for every subsample and the mean of the 10 results was estimated as the misclassification risk value [30]. In addition, the accuracy of the DT and multiple logistic analyses was compared [31]. 

### 4.7. Ethics

This study was carried out in accordance with Ethical Guidelines for Medical and Health Research Involving Human Subjects. The protocol was approved by the ethics committee of the Hokkaido University Hospital (study protocol no. 019-0034), JCHO Sapporo Hokushin Hospital (study protocol no. 2019-08), Sunagawa City Medical Center (study protocol no. 2019-14), and Sapporo City General Hospital (study protocol no. R01-059-600). 

## 5. Conclusions

We successfully constructed a simple flowchart predicting the VCM-induced AKI at the time of initiating VCM administration. This flowchart had a higher validity, than that of the model proposed in our previous studies [17,19], because of the implementation of a multicenter study and targeting of patients who were administered a standard VCM dose. Using this flowchart constructed by DT analysis, clinicians and pharmacists can easily evaluate the risk of AKI, by visual observation. Our flowchart has potential utility as a tool for deciding a treatment policy for patients who require VCM therapy.

## Figures and Tables

**Table 1:** Comparison of patient’s characteristics between those with AKI and those without.

Characteristics	With AKI	Without AKI	*p-*Value
	(n = 115)	(n = 728)	
Age (years), median (Q1–Q3)	69.2 (62.8–77.0)	69.6 (62.0–77.4)	0.796 ^c^
Male/female, n/n (%/%)	72/43 (62.6/37.4)	472/256 (64.8/35.2)	0.643 ^a^
Height (cm), median (Q1–Q3)	161.6	161	0.533 ^c^
	(153.8–168.0)	(153.0–167.0)	
Body weight (kg), median (Q1–Q3)	58.7 (50.6–67.8)	55.1 (47.7–64.0)	0.016 ^c,^*
BMI (kg/m^2^), median (Q1–Q3)	22.5 (20.1–25.1)	21.7 (19.1–24.4)	0.015 ^c,^*
≥ 30, n (%)	9 (7.83)	29 (3.98)	0.065 ^a^
BUN (mg/dL), median (Q1–Q3)			
Initial administration	26.0 (15.0–36.0)	18.0 (13.0–25.0)	<0.001 ^c,^*
Initial TDM	32.0 (19.7–49.2)	16.0 (11.0–22.4)	<0.001 ^c,^*
Maximum	38.9 (23.3–54.3)	18.0 (13.0–26.0)	<0.001 ^c,^*
Scr (mg/dL), median (Q1–Q3)			
Initial administration	0.78 (0.65–1.17)	0.76 (0.60–0.97)	0.024 ^c,^*
Initial TDM	1.21 (0.82–1.66)	0.70 (0.56–0.87)	<0.001 ^c,^*
Maximum	1.54 (1.20–1.99)	0.80 (0.64–1.01)	<0.001 ^c,^*
Adjusted CCr (mL/min), median (Q1–Q3)	63.7 (42.3–82.3)	66.6 (47.8–84.0)	0.342 ^c^
< 86.6, n (%)	92 (80.0)	577 (79.3)	0.855 ^a^
VCM daily dose (mg), median (Q1–Q3) Initial administration After initial TDM	1500 (1000–2000) 1225 (750–2000)	1500 (1000–2000) 1500 (1000–2000)	0.416 ^c^ <0.001 ^c,^*
Duration of therapy (days), median (Q1–Q3)	7 (4–11)	7 (5–11)	0.038 ^c,^*
Days to initial TDM (days), median (Q1–Q3)	3 (3–4)	4 (3–4)	0.683 ^c^
Initial VCM trough concentration (mg/L), median (Q1-Q3)	15.5 (10.8–21.3)	10.9 (8.14–14.3)	<0.001 ^c,^*
With loading dose, n (%)	24 (20.9)	149 (20.5)	0.921 ^a^
Loading dose (mg), median (Q1–Q3)	1000 (1000–1000)	1000 (1000–1000)	0.342 ^c^
Residence in ICU, n (%)	34 (29.6)	113 (15.5)	<0.001 ^a,^*
Type of infection of infective endocarditis, n (%)	2 (1.74)	9 (1.24)	0.659 ^a^
Underlying disease			
Heart disease, n (%)	14 (12.2)	43 (5.91)	0.013 ^a,^*
Diabetes, n (%)	31 (27.0)	162 (22.3)	0.265 ^a^
Hypertension, n (%)	55 (47.8)	204 (28.0)	0.594 ^a^
Cancer, n (%)	47 (40.9)	327 (44.9)	0.417 ^a^
Concomitant medications, n (%)			
NSAIDs	36 (31.3)	222 (30.5)	0.861 ^a^
Furosemide	37 (32.2)	128 (17.6)	<0.001 ^a,^*
Aminoglycosides	2 (1.74)	8 (1.10)	0.634 ^b^
AMPH-B	5 (4.35)	3 (0.41)	0.002 ^b,^*
Vasopressor drugs	32 (27.8)	55 (7.55)	<0.001 ^a,^*
TAZ/PIPC	26 (22.6)	93 (12.8)	0.005 ^a,^*
Nitric acid-based medicine	8 (6.96)	40 (5.49)	0.530 ^a^
Tacrolimus	6 (5.22)	29 (3.98)	0.461 ^a^

Abbreviations: AKI, acute kidney injury; Q1, first quartile; Q3, third quartile; BMI, body mass index; VCM, vancomycin; BUN, blood urea nitrogen; Scr, serum creatinine; CCr, creatinine clearance; TDM, therapeutic drug monitoring; ICU, intensive care unit; NSAIDs, non-steroidal anti-inflammatory drugs; AMPH-B, amphotericin B; TAZ/PIPC, tazobactam/piperacillin. ^a^ Pearson’s chi-squared test, ^b^ Fisher’s exact test, and ^c^ Mann–Whitney *U* test. * *p* < 0.05.

**Table 2:** Logistic regression analysis (dependent variable: AKI).

	Univariate Analysis			Multiple Analysis		
Independent Variable	OR	OR (95% CI)	*p*-Value	OR	OR (95% CI)	*p*-Value
Type of infection of infective endocarditis	1.41	0.30–6.63	0.660	-	-	-
**Underlying disease**						
Heart disease	2.21	1.17–4.18	0.015 ^☨^	1.54	0.77–3.07	0.227
Diabetes	1.29	0.82–2.02	0.265	-	-	-
Hypertension	1.12	0.73–1.73	0.594	-	-	-
Cancer	0.85	0.57–1.26	0.417	-	-	-
**Concomitant medications**						
NSAIDs	1.04	0.68–1.59	0.861	-	-	-
Furosemide	2.22	1.44–3.44	0.001 ^☨^	1.74	1.09–2.76	0.021 *
Aminoglycosides	1.59	0.33–7.60	0.559	-	-	-
AMPH-B	10.99	2.59–46.61	0.001 ^☨^	9.32	2.07–41.71	0.004 *
Vasopressor drugs	4.78	2.89–7.72	<0.001 ^☨^	3.68	2.20–6.18	<0.001 *
TAZ/PIPC	2.00	1.22–3.25	0.006 ^☨^	1.73	1.03–2.91	0.038 *
Nitric acid-based medicine	1.29	0.59–2.82	0.531	-	-	-
Tacrolimus	1.33	0.54–3.27	0.539	-	-	-
BMI ≥ 30	2.05	0.94–4.44	0.070 ^☨^	1.95	0.87–4.39	0.105
CCr < 86.6 mL/min	1.05	0.64–1.71	0.855	-	-	-

Abbreviations: AKI: acute kidney injury, OR: odds ratio, 95% CI: 95% confidence interval, NSAIDs: non-steroidal anti-inflammatory drugs, AMPH-B: amphotericin B, TAZ/PIPC: tazobactam/piperacillin, BMI: body mass index, CCr: creatinine clearance. ^☨^: *p* < 0.2 were included in the multiple analysis. * *p* < 0.05 were considered statistically significant.

**Table 3:** Comparison of standard doses.

	Thomson et al. [21]		Present Study
CCr (mL/min)	Single Dose (mg)	Dosing Interval (h)	Standard Dose
<20	500	48	250–750 mg/48 h
20–29	500	24	250–750 mg/day
30–39	750	24	500–1000 mg/day
40–54	500	12	500–1500 mg/day
55–74	750	12	1000–2000 mg/day
75–89	1000	12	1500–2500 mg/day
90–110	1250	12	2000–3000 mg/day
>110	1500	12	2500–3500 mg/day

Abbreviations: CCr: Creatinine clearance.
